# Expanding Medical Student Knowledge and Impacting Patient Outcomes Through a Student-Run Clinic: A Case-Based Reflection

**DOI:** 10.7759/cureus.39532

**Published:** 2023-05-26

**Authors:** Andrew J Ordille, Gianna Antinori, Cyrus Mowdawalla, John McGeehan

**Affiliations:** 1 Department of Biomedical Sciences, Cooper Medical School of Rowan University, Camden, USA

**Keywords:** stroke care, cryptogenic strokes, primary care medicine, integrated medical education, access to healthcare, student-run clinics

## Abstract

Cryptogenic stroke is a debilitating condition that requires follow-up care and treatment that is appropriate for the underlying etiology. Here, we present the case of a 46-year-old uninsured patient with an undocumented immigration status who presented to our student-run clinic (SRC) for the management of her post-stroke care. She initially presented to an outside hospital with focal neurological deficits, was diagnosed with an acute stroke, and was told to follow up with a primary care provider. The patient established care at the Cooper Medical School of Rowan University’s SRC one week following her stroke event. The SRC served as a conduit for access to healthcare services necessary for her recovery and secondary prevention of future strokes which otherwise would have been unattainable due to the patient’s socioeconomic constraints. These services and treatments included specialist appointments, anticoagulation medications, physical and speech therapy, labs, placement of an internal heart rhythm monitor, and surgical closure of a patent foramen ovale. All services, medications, and procedures were provided free of charge. One year following her stroke, the patient is living without disability and has had no recurrence of a cerebrovascular ischemic event. This case highlights the dual-purposed value of SRCs in providing both meaningful clinical educational experiences to students and necessary health care to disadvantaged patients.

## Introduction

Stroke is a major cause of mortality and morbidity in the United States [1]. Although primarily seen in elderly patients, stroke can affect younger patients through cardioembolic phenomena, such as patent foramen ovale (PFO). The persistence of this anatomical conduit is a common finding seen in up to 25% of the general population, with a strong association observed between embolic events and younger patients [2,3]. PFOs account for approximately 40% of all cryptogenic strokes and transient ischemic attacks in patients less than 55 years of age [1,4].

Significant healthcare dollars are spent investigating the causes of cerebral vascular accidents (CVAs), providing secondary prevention, and rehabilitating functional abilities. Reports estimate the average cost of outpatient stroke rehabilitation services and medications in the first year following a stroke to be approximately $17,000 in the United States [5].

Underserved and uninsured communities, like Camden, New Jersey, face significant barriers to these healthcare services. United States census data from 2005 reported Camden to be the poorest city in America, with nearly half of the city’s population living below the federal poverty line and many residents uninsured [6]. The Cooper Rowan Clinic is a student-run clinic (SRC) through Cooper University Hospital’s affiliated medical school, the Cooper Medical School of Rowan University, that offers free medical treatment and rehabilitation services for these at-risk patients in Camden, New Jersey.

The Cooper Rowan Clinic, similar to many other SRCs, allows integrated medical student teams (i.e., comprising first-, second-, and third-year medical students) to serve as the primary care provider for uninsured and undocumented patients of Camden, New Jersey, under the direct supervision of a precepting physician at the SRC. Student teams provide longitudinal care for their patients across their medical education for medical issues, including diabetes, hypertension, and mental health conditions. Here, students are responsible for obtaining a patient’s history, performing a physical examination, and formulating an assessment and plan which is presented to and collaboratively influenced by the supervising physician. Through the financial support of Cooper University Hospital, the SRC care teams can provide referrals for laboratory tests, imaging, specialist appointments, procedures, medications, and various therapy services within the Cooper University Hospital healthcare network free of charge. Many SRCs, like the Cooper Rowan Clinic, adopt a model where patient visits are extended (i.e., exceeding one hour) to allow more comprehensive visits for patients and an optimal learning environment for medical students. However, individual SRCs vary in their capacity and reach, with some SRCs unable to offer the comprehensive care that other SRCs provide and with limitations to the patient population being served (i.e., insurance status and geographic area of residence) [7].

Universally, SRCs have benefited medical students through early patient experiences, clinical education, and fostering a sense of service to the community. More importantly, these free health clinics provide accessible health care to medically underserved patients who otherwise would not have been able to receive necessary treatments [7]. Here, we present a case of cryptogenic stroke in a young, uninsured patient, whose outpatient management at our SRC resulted in the discovery of the stroke source, medical coverage of necessary therapeutic intervention, and recovery through rehabilitative services.

## Case presentation

**Figure 1:**
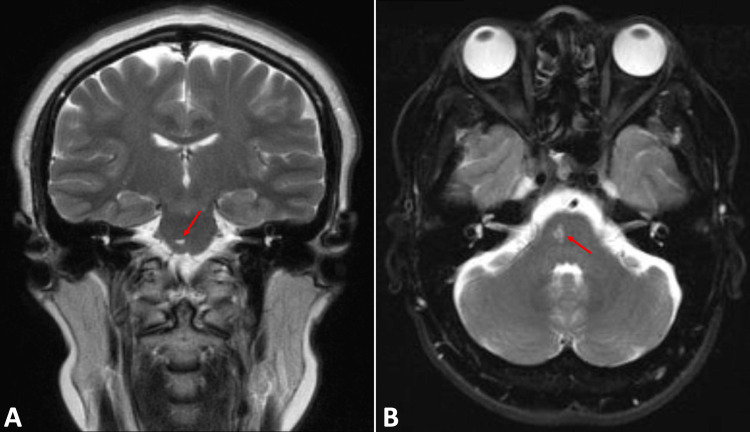
MRI of the brain without contrast demonstrating a small focal lobulated hyperintense T2 focus of the right pons measuring approximately 8 mm × 4 mm × 2 mm in coronal (A) and axial views (B). MRI = magnetic resonance imaging

A 46-year-old uninsured, undocumented female with a history of hyperlipidemia presented to an outside hospital with pre-syncopal episodes and resultant left-sided weakness and numbness. Neurologic examination revealed diminished sensation to the left side of the face, arm, and leg. Upon arrival at the Emergency Department (ED), the patient’s vital signs, troponins, D-dimer, and electrocardiogram were normal. Although computed tomography and computed tomography angiography imaging of the head and neck imaging were negative for any vascular or malignant pathologies, the magnetic resonance imaging of the brain revealed an acute pontine infarct right of the midline with no evidence of hemorrhage (Figure 1).

**Figure 2:**
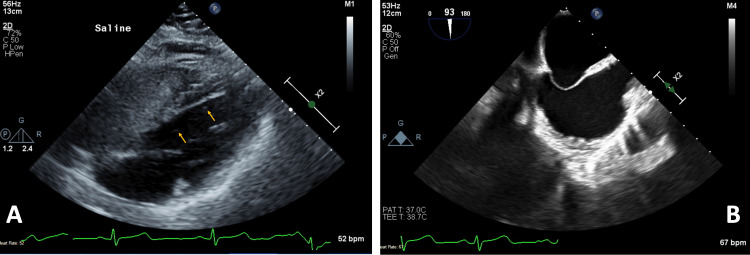
TEE demonstrating (A) crossover of bubbles (yellow arrows) on an agitated saline study from the right heart to the left heart within three cardiac cycles, indicative of an intracardiac shunt; and (B) a bicaval view of the interatrial septal aneurysm. TEE = transesophageal echocardiogram

As the patient presented more than 4.5 hours from symptom onset, tissue plasminogen activator (tPA) was withheld. A transthoracic echocardiogram revealed the presence of an atrial septal aneurysm and PFO with right-to-left shunting on bubble injection (Figure 2). The patient was discharged on statin therapy and dual antiplatelet therapy and established care with our SRC the following week.

**Figure 3:**
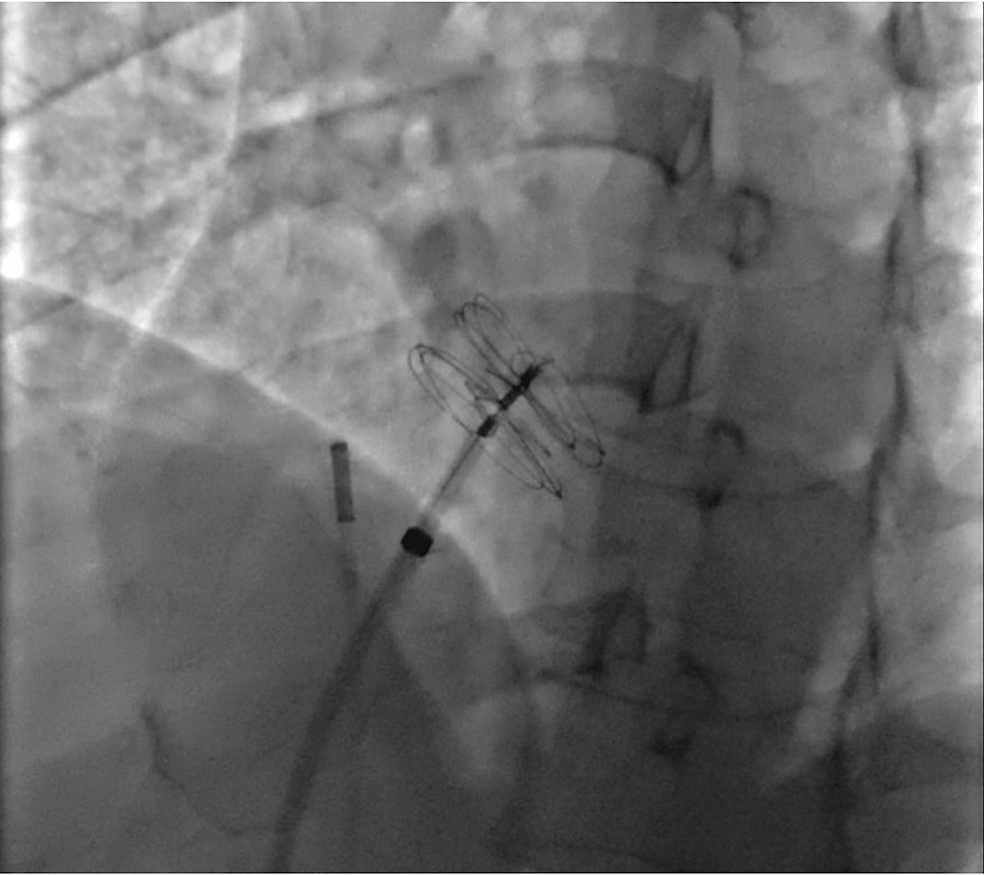
Intraprocedural fluoroscopy demonstrating the deployment of PFO occlusion device via cardiac catheterization. PFO = patent foramen ovale

With the oversight of the SRC, the patient was provided referrals for ongoing evaluation by subspecialists, including neurologists, hematologists, and cardiologists. A hematologic workup demonstrated no evidence of a hypercoagulable state which might have contributed to her presentation. Multiple appointments with cardiology resulted in anatomy scans, intracardiac rhythm monitoring, and PFO closure. Referrals to these subspecialty clinics, a procedure by interventional cardiologists, and therapy services (i.e., physical and speech therapies) were initiated free of charge (Figure 3). Following closure and six months of medical and rehabilitative services, the patient’s motor and sensory abnormalities returned to baseline. She returned to work and is currently living without disability one year following her CVA.

## Discussion

**Figure 4:**
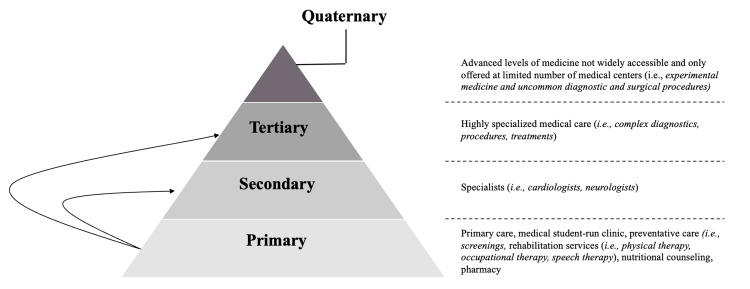
Pyramid structure representing the levels and domains of healthcare practice within broad healthcare systems. The tiers represent increasing degrees of specialization and healthcare costs, as explained by Gosadi et al. [9]. The greatest number of patients are seen at the first level and with diminished numbers seen in higher levels of specialized care. In this case report, a medical student-run clinic provided access to higher levels of care which was otherwise unattainable.

In this case, timely treatments with medication and referrals to specialists, surgical repair of the PFO, and placement of an internal heart rhythm monitor were crucial. The patient’s risk of paradoxical embolism score of 8 suggests an 84% chance that her stroke was attributable to her PFO, while also citing a two-year recurrence risk of 6% [8]. For our patient to have achieved full recovery, multiple levels of healthcare services were required (i.e., primary, secondary, and tertiary levels of care) (Figure 4). The SRC was the patient’s primary level of service by establishing longitudinal care. The SRC served as a vital adjunct to higher levels of care (i.e., secondary and tertiary) by facilitating access to services otherwise unattainable due to the patient’s socioeconomic constraints.

The SRC provides medical students with the guided responsibility and opportunity to not only improve clinical skills but to also understand how to navigate the healthcare system as future providers. Our team faced challenges in advocating for our patient to be seen by multiple specialists to obtain the correct diagnosis. By spearheading our patient’s care, we were able to improve our patient’s health literacy and increase her confidence to navigate the healthcare system effectively. The encounter highlighted the importance of patient advocacy and peer-to-peer education in providing affordable and personalized patient-centered care.

Unfortunately, there is very limited interaction with the healthcare system beyond the walls of the structured medical school curriculum. Many medical schools have addressed this need through the implementation of health systems science (HSS) courses, which explore tenets such as patient-centered care, healthcare policy and payment, social determinants of health, and system improvement [10]. SRCs allow students to employ HSS principles by advocating for diverse patient populations in clinical settings.

SRCs are becoming more prevalent among medical schools, with over 75% of United States medical schools operating an SRC [11]. Adequate funding from the state and hospital systems is vital for SRCs to exist. Estimates from the Indiana University Student Outreach Clinic provided over $150,000 of free healthcare in one year to their community [12]. Funding SRCs help decrease inappropriate ED utilization and increase value-based health care in both clinical settings [13].

With the increasing cost of health care and lack of primary care access, we call for all United States medical schools to establish SRCs. The Association of American Medical Colleges projects a shortage of 21,000-55,000 primary care physicians in the United States by 2033 [14]. This shortage will likely exacerbate primary care inaccessibility for at-risk patient populations. SRCs provide vital care to patients with socioeconomic barriers with efficient utilization of healthcare dollars but may also encourage medical students to pursue primary care through early exposure to the field.

## Conclusions

Stroke is a medical condition that is particularly debilitating for patients of an uninsured healthcare status due to disproportionate access to necessary healthcare services. This case highlights the much-needed intervention of an SRC for a patient with an increased recurrence risk for stroke who otherwise would not have been able to receive care. In this situation, the SRC acted as a primary care provider which allowed the patient to access necessary higher levels of care. This case also highlights the dual-purposed value of SRCs in providing both meaningful clinical educational experiences and necessary health care to disadvantaged patients in a leading tertiary healthcare system. We advocate that SRCs should be utilized by all medical education institutions. Furthermore, SRCs may serve as a solution to addressing the projected primary care provider shortage by increasing student exposure to the field.
